# Structural Study of Cell Attachment Peptide Derived from Laminin by Molecular Dynamics Simulation

**DOI:** 10.1371/journal.pone.0149474

**Published:** 2016-02-18

**Authors:** Hironao Yamada, Sakiko Mori, Takeshi Miyakawa, Ryota Morikawa, Fumihiko Katagiri, Kentaro Hozumi, Yamato Kikkawa, Motoyoshi Nomizu, Masako Takasu

**Affiliations:** 1 School of Life Sciences, Tokyo University of Pharmacy and Life Sciences, 1432–1 Horinouchi, Hachiouji, Tokyo 192–0392, Japan; 2 School of Pharmacy, Tokyo University of Pharmacy and Life Sciences, 1432–1 Horinouchi, Hachiouji, Tokyo 192–0392, Japan; University of Akron, UNITED STATES

## Abstract

Peptides with cell attachment activity are beneficial component of biomaterials for tissue engineering. Conformational structure is one of the important factors for the biological activities. The EF1 peptide (DYATLQLQEGRLHFMFDLG) derived from laminin promotes cell spreading and cell attachment activity mediated by α2β1 integrin. Although the sequence of the EF2 peptide (DFATVQLRNGFPYFSYDLG) is homologous sequence to that of EF1, EF2 does not promote cell attachment activity. To determine whether there are structural differences between EF1 and EF2, we performed replica exchange molecular dynamics (REMD) simulations and conventional molecular dynamics (MD) simulations. We found that EF1 and EF2 had β-sheet structure as a secondary structure around the global minimum. However, EF2 had variety of structures around the global minimum compared with EF1 and has easily escaped from the bottom of free energy. The structural fluctuation of the EF1 is smaller than that of the EF2. The structural variation of EF2 is related to these differences in the structural fluctuation and the number of the hydrogen bonds (H-bonds). From the analysis of H-bonds in the β-sheet, the number of H-bonds in EF1 is larger than that in EF2 in the time scale of the conventional MD simulation, suggesting that the formation of H-bonds is related to the differences in the structural fluctuation between EF1 and EF2. From the analysis of other non-covalent interactions in the amino acid sequences of EF1 and EF2, EF1 has three pairs of residues with hydrophobic interaction, and EF2 has two pairs. These results indicate that several non-covalent interactions are important for structural stabilization. Consequently, the structure of EF1 is stabilized by H-bonds and pairs of hydrophobic amino acids in the terminals. Hence, we propose that non-covalent interactions around N-terminal and C-terminal of the peptides are crucial for maintaining the β-sheet structure of the peptides.

## Introduction

Peptides are functional molecules that can have various biological activities, and some peptides, which are part of an original protein, can mimic the functions of the original protein. In tissue engineering, peptides having cell attachment activity are useful as adhesive agents for artificial extracellular matrices, and peptides have been employed as biomaterial components [1, 2]. Hozumi et al. also reported that the peptides derived from laminin have the potential to be developed as useful biological materials [3].

Laminin is a giant glycoprotein (molecular weight of around 500–900 kDa) that consists of three subunits (α, β, and γ chains). It has diverse biological activities such as the promotion of cell attachment, cell migration, tumor metastasis, neurite outgrowth and angiogenesis [4–8]. Five types of α chains, three types of β chains and three types of γ chains have been found, respectively. For laminin, 19 isoforms (laminin-1 to 19) have been isolated, and these isoforms are found in diverse tissues [8–16]. Among these isoforms, laminin-1, which consists of three subunits (α1 chain, β1 chain and γ1 chain), was discovered first [5], and it enhances diverse biological activities, such as cell attachment and cell migration [17, 18]. A number of biomolecules (integrin, syndecan and others) are identified as laminin-1 receptors [19]. Nomizu et al. identified several bioactive peptides that reproduce the function of part of the laminin-1 [20–23].

**Fig 1:**
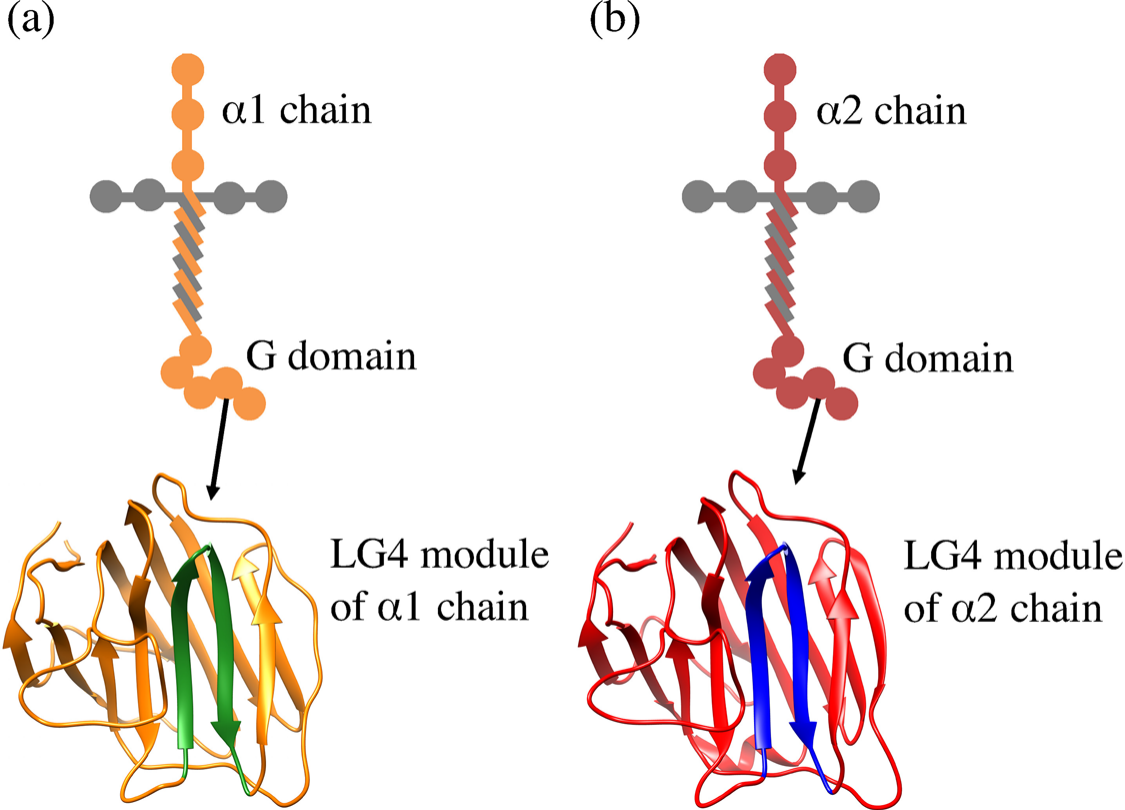
Positions of EF1 and EF2 peptides in laminin. (a) and (b) are positions of EF1 and EF2 peptide in mouse laminin α1, 2 chain LG4 module. Each site showed by green and blue color has the same sequence as EF1 and EF2, respectively.

Laminin α (α1–α5) chains also have diverse biological activities. In the C-terminal region of laminin α chains, G domain (globular domain) consisting of five laminin G domain-like modules (LG1–5) exists. The G domain plays an important role in the biological activities of the α chains, and several sequences with biological activities are identified [24–29]. Each LG module (LG1–5) forms a 14-stranded β-sheet (A–N strands) sandwich structure [30]. Previously, Suzuki et al. identified several active peptides (EF1, EF2, EF3, EF4 and EF5) that exist in the loop region of the LG4 module [31]. These peptides have chain-specific sequences, and each of these peptides interacts with specific receptor (α2β1 integrin and syndecan) [31]. One of these peptides, EF1 peptide (DYATLQLQEGRLHFMFDLG, mouse laminin α1 chain residues 2747–2765 [32]), is located on the loop region of the E and F strands of the LG4 module in the α1 chain as shown in Fig 1A, and EF1 promotes cell spreading and cell attachment mediated by the α2β1 integrin [31, 33]. Suzuki et al. also determined that the EF1Xm peptide (LQLQEGRLHFXFD, X: norleucine) is the minimal sequence required for cell attachment activity [31]. The cell attachment activity of EF1 was evaluated using human neonatal dermal fibroblasts (HDFs), human fibrosarcoma cells (HT1080) and human submandibular glands (HSG) cells in vitro. Moreover, it was reported that cyc-EF1Xm (CLQLQEGRLHFXFDC, X: norleucine), obtained by cross-linking EF1Xm via disulfide bonding, displayed more cell attachment activity than the corresponding EF1 [31]. These results suggest that the biological activity of the EF1 depends on the hairpin-like structure, and that the cyclization of linear peptide may be important for biological activities. On the other hand, the EF2 peptide (DFATVQLRNGFPYFSYDLG, mouse laminin α2 chain residues 2808–2826), which is a homologous sequence of EF1, is located on the loop region of the E and F strands of the LG4 module in the laminin α2 chain [33] as shown in Fig 1B. Although homologous sequences usually have the same functions, EF2 does not have cell attachment activity [33]. The cell attachment activity of EF2 was also evaluated using HDFs in vitro, as well as EF1. Therefore, it is worth investigating whether the steric structures of EF1 and EF2 are related to this difference in cell attachment activity.

Both EF1 and EF2 display a β-sheet structure in the LG4 module of the mouse laminin α1 and 2 chain, determined by an X-ray diffraction (PDB ID: 2JD4 [32] and PDB ID: 1DYK [34]). In our previous research [35, 36], we performed simulated annealing to investigate the structures of EF1 and EF2 for the global minimum of potential energy. As the initial structure, we used the part of EF2 that was derived from the structure of the mouse laminin 2 chain LG4 module determined by an X-ray diffraction. We also used the peptide obtained by amino acid substitution of EF2 as the initial structure of the EF1. In the simulated annealing, we showed that EF1 had a hairpin-like structure, whereas EF2 did not have such structure in water solution. In this study, we use replica exchange molecular dynamics (REMD) simulation [37], which is known as one of the structure sampling methods, to sample the structures of EF1 and EF2 extensively. As the initial structure of EF1, we used the part of EF1 of the crystal structures (mouse laminin α1 chain LG4 modules) determined by an X-ray diffraction. To specify the dynamical behaviors of EF1 and EF2, we also needed to investigate them in water. We perform molecular dynamics (MD) simulations of EF1 and EF2 at 300 K and 1 bar in an NPT ensemble. Moreover, we analyze the results obtained from the simulations, focusing on non-covalent interactions.

## Materials and Methods

### Data preparation of peptide sequence

As the initial structure, we obtained the coordinates of EF1 and EF2 peptide from the crystal structures (mouse laminin α1 and 2 chain LG4 modules) determined by an X-ray diffraction. The PDB IDs of the crystal structures are 2JD4 [32] and 1DYK [34], respectively. Both EF1 and EF2 were capped with acetyl (Ace) and N-methyl (Nme) groups at the N- and C-termini, respectively, as shown in Table 1. Henceforth, we refer to the initial structures of EF1 and EF2 as the crystal structure.

**Table 1:** Amino acid sequences of EF1 and EF2 peptides.

Peptides	Amino acid sequences
EF1	Ace-DYATLQLQEGRLHFMFDLG-Nme
EF2	Ace-DFATVQLRNGFPYFSYDLG-Nme

### Molecular dynamics simulation

We used GROMACS version 5.1 [38] for REMD simulations and version 4.6.6 [38] for conventional MD simulations. GROMACS is an application software package of molecular dynamics. We used AMBER99SB-ildn all-atom force field [39] for peptides in the MD simulations, and we employed TIP3P water model [40] as the solvent. The time step was set at 2.0 fs. For constraint algorithm, we used the Linear Constraint Solver (LINCS) algorithm [41] to fix the lengths or the angles of the bonds including hydrogen. We used the Particle Mesh Ewald method [42] for the computational algorithm of long-range electrostatic interaction. For temperature and pressure coupling, we used the Nosé-Hoover and Parrinello-Rahman method, respectively. The above-mentioned conditions were set for both REMD and conventional MD simulations.

#### Replica exchange molecular dynamics (REMD)

Replica exchange molecular dynamics (REMD) simulation is a method that allows efficient sampling of peptide structure. In accordance with the REMD method [37], we prepared 48 replicas (300–450.5 K) with intervals of 2.5–4 K for EF1 and EF2 peptides, respectively, as follows. The temperature for each replica is shown in Table 2. We placed each peptide in a system containing 7,953 (for EF1) or 7,011 (for EF2) water molecules (TIP3P water model) under periodic boundary conditions. We performed equilibrium simulation at 300 K and at 1 bar for 1 ns. The system after the equilibrium simulation was employed as the initial state for each replica. We next performed pre-simulations at constant temperature and at constant volume for each replica to set it at specified temperatures. Equilibrating each replica at its own temperature, we simulated each replica independently at constant temperature and at constant volume for 60 ns. The exchange frequency between replicas was set at time step of 2.0 ps. In the analysis, we used trajectories from 5 to 60 ns obtained by runs of each replica.

**Table 2:** Temperature of each replica.

Replica 1	300 K	Replica 18	346 K	Replica 35	402 K
2	302.5 K	19	349 K	36	405.5 K
3	305 K	20	352 K	37	409 K
4	307.5 K	21	355 K	38	412.5 K
5	310 K	22	358 K	39	416 K
6	312.5 K	23	361 K	40	419.5 K
7	315 K	24	364 K	41	423 K
8	317.5 K	25	367 K	42	426.5 K
9	320 K	26	370.5 K	43	430.5 K
10	322.5 K	27	374 K	44	434.5 K
11	325 K	28	377.5 K	45	438.5 K
12	328 K	29	381 K	46	442.5 K
13	331 K	30	384.5 K	47	446.5 K
14	334 K	31	388 K	48	450.5 K
15	337 K	32	391.5 K		
16	340 K	33	395 K		
17	343 K	34	398.5 K		

#### Conventional molecular dynamics (MD) simulation

In the conventional MD simulations, EF1 and EF2 were placed in a cubic box containing 7,118 and 7,100 water molecules (TIP3P water model), respectively, under periodic boundary conditions. First, we performed energy minimization using the steepest descent method [43]. Second, we set the molecules in equilibrium for 1 ns at constant temperature (300 K) and for 50 ns at constant pressure (1 bar) ensemble as pre-simulations. Finally, we performed simulations for 2 μs at constant temperature (300 K) and constant pressure (1 bar), and EF1 and EF2 were simulated twice using different values of initial positions and initial velocities of water molecules.

### Autocorrelation function

One of methods to investigate the accuracy of REMD is the calculation of the autocorrelation function [44, 45]. We calculated the autocorrelation function *C(t)* based on following equation,

$$C(t)=\frac{1}{N-j}\sum_{i=0}^{N-1-j}f(i\Delta t)f((i+j)\Delta t),$$ (1)

where *t* = *j*Δ*t*, *f* is a physical quantity, and *N* is the number of the frames for the physical quantity in the simulation. For the autocorrelation function, we choose the rate of formation of β-sheet structure because EF1 and EF2 have β-sheet structure in laminin. The calculation of the secondary structure is based on the dssp program [46] to perform secondary structure assignment for each residue of peptides and proteins.

### Free energy landscape

The free energy landscape of the system was calculated on the ground of the probability distribution of structures obtained from the simulations. For molecular simulation, Gibbs free energy change Δ*G* was applied to show the stability and the state of the system. It was also used in the energy analysis of folding and chemical reaction. In this study, we calculate the free energy landscape to investigate the variety of EF1 and EF2 conformations obtained from REMD and conventional MD simulations. The relative Gibbs free energy change Δ*G* is defined as follows [47],

$$\Delta G=k_{B}T\log\frac{P\left(x,y\right)}{P_{\max}},$$ (2)

where *k*_B_ is the Boltzmann constant, *T* is temperature of the system in the simulation, and *P*(*x*,*y*) is the probability distribution function at the reaction coordinates area, where *x* and *y* are the reaction coordinates. *P*_max_ is the maximum probability distribution in the sampled area mapped from sampled trajectories in the simulations, where Δ*G* = 0 for the lowest free energy. We mapped the free energy landscape on the plane of the root mean square deviation (RMSD) and radius of gyration (*R*_g_), as reaction coordinates. For the calculation of RMSD, we fitted the structure to minimize the value of RMSD with respect to the reference structure by rotating the entire peptide structure.

### Calculation of hydrogen bonds

Hydrogen bonds (H-bonds) are important for the formation of secondary structures (β-sheets and α-helices) and are observed between the backbone oxygen and amide hydrogen in the the secondary structure of peptides and proteins. The β-sheets contain H-bonds between N-H and C = O in the peptide bond of adjacent chains. We calculated the length and the number of the H-bonds, and the positions of the H-bonds for EF1 and EF2 are shown in the Table 3. These values are quantities that characterize the stability of the β-sheet structure.

**Table 3:** Positions of hydrogen bonds (H-bonds), characterizing β-sheet structures of EF1 and EF2. Each of the H-bonds is defined as HB1-HB8.

Item	Pairs of H-bonds (EF1)			Pairs of H-bonds (EF2)		
HB1	TYR_2_–NH	•••	OC–ASP_17_	PHE_2_–NH	•••	OC–ASP_17_
HB2	TYR_2_–CO	•••	HN–ASP_17_	PHE_2_–CO	•••	HN–ASP_17_
HB3	THR_4_–NH	•••	OC–MET_15_	THR_4_–NH	•••	OC–SER_15_
HB4	THR_4_–CO	•••	HN–MET_15_	THR_4_–CO	•••	HN–SER_15_
HB5	GLN_6_–NH	•••	OC–HIS_13_	GLN_6_–NH	•••	OC–TYR_13_
HB6	GLN_6_–CO	•••	HN–HIS_13_	GLN_6_–CO	•••	HN–TYR_13_
HB7	GLN_8_–NH	•••	OC–ARG_11_	ARG_8_–NH	•••	OC–PHE_11_
HB8	GLN_8_–CO	•••	HN–ARG_11_	ARG_8_–NH	•••	OC–PHE_11_

## Results

### Structure sampling by replica exchange molecular dynamics simulation

**Fig 2:**
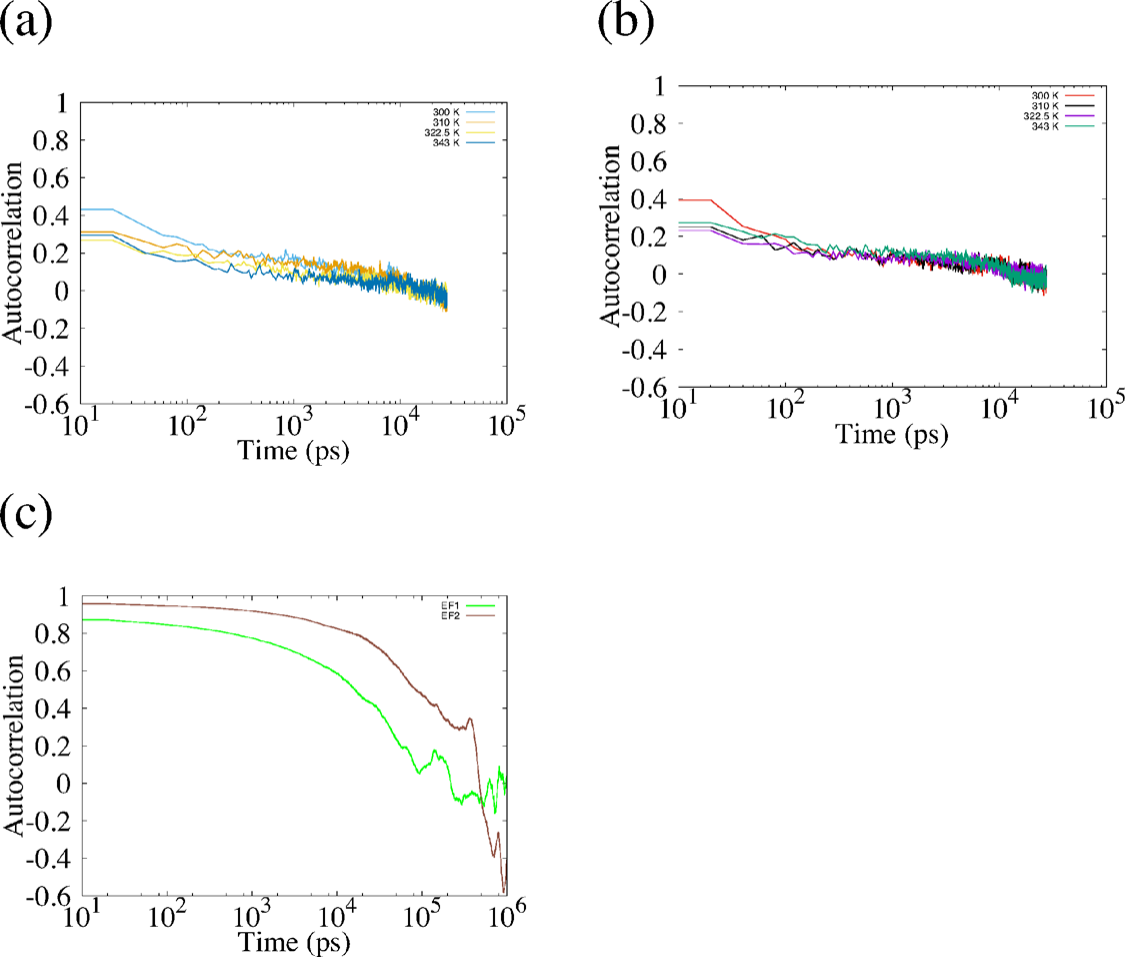
Autocorrelation of rate of formation of β-sheet structure. (a) and (b) show autocorrelation of rate of formation of β-sheet structure obtained from replicas of EF1 and EF2 used for analysis. (c) shows the autocorrelation of EF1 and EF2 obtained from conventional MD.

We applied the REMD simulation to the EF1 and EF2 and confirmed the accuracy of REMD simulation by autocorrelation function of rate of formation of β-sheet structure. The autocorrelation function is shown in Fig 2, and we show the average rate of formation of β-sheet structure in Table 4. The autocorrelation function for REMD rapidly decreased compared with the autocorrelation function for the conventional MD simulation. In Table 4, the difference between the average rate of REMD and that of conventional MD is small. However, the variation of the rate of formation is larger for REMD than for conventional MD. These results show that the dependence of initial structure rapidly disappeared in REMD, and that the structures of EF1 and EF2 are sampled well by REMD.

**Table 4:** Average rate of formation of β-sheet structure.

		REMD	Conventional MD
EF1	300 K	0.444 (0.165)	0.438 (0.068)
	310 K	0.439 (0.165)	
	322.5 K	0.412 (0.169)	
	343 K	0.382 (0.193)	
EF2	300 K	0.415 (0.212)	0.471 (0.148)
	310 K	0.390 (0.218)	
	322.5 K	0.342 (0.217)	
	343 K	0.310 (0.213)	

Values in parentheses indicate the standard deviation.

**Fig 3:**
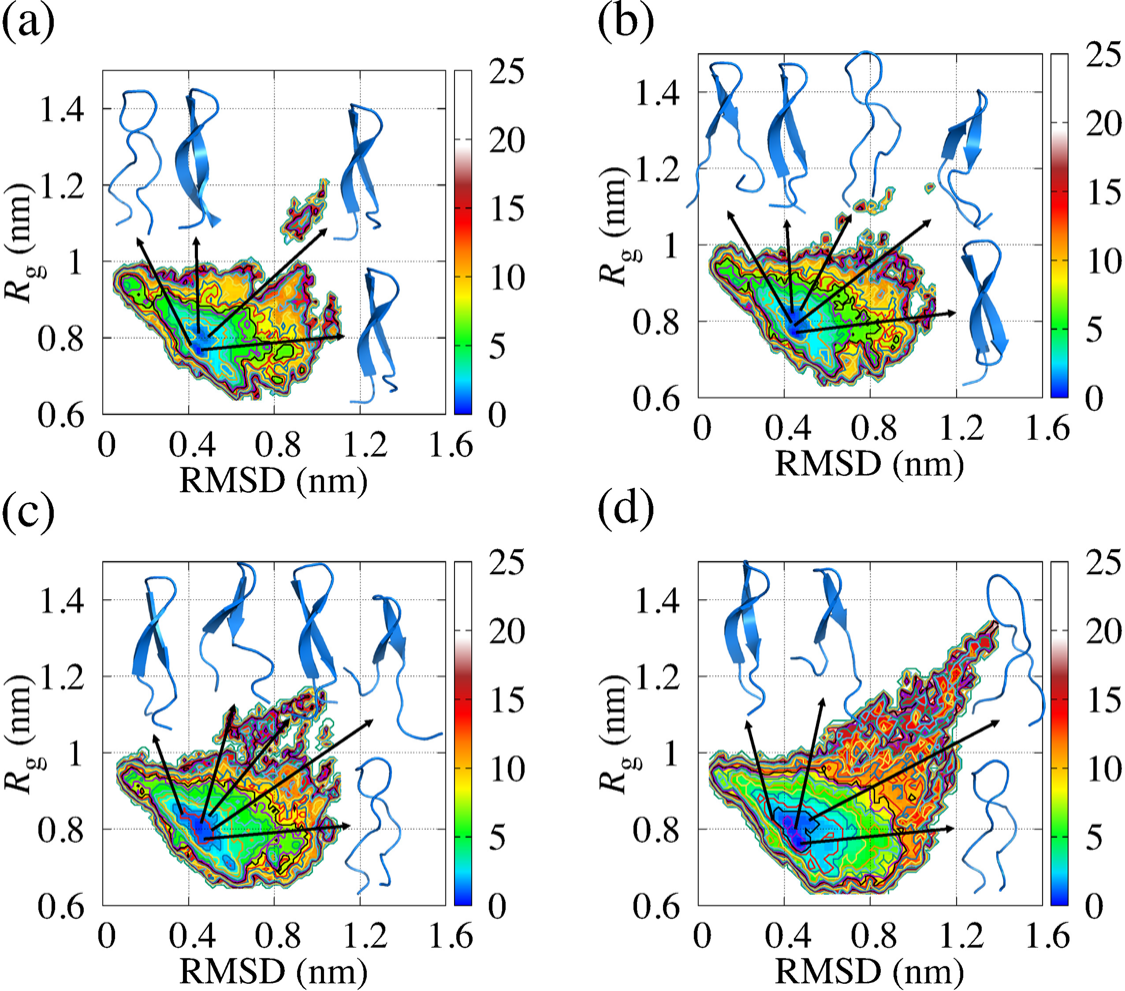
Free energy landscape of EF1 obtained from REMD. These maps indicate the existence probabilities of the EF1 peptide obtained from simulations at 300 K (a), 310 K (b), 322.5 K (c) and 343 K (d). Relative free energy change (kJ/mol) is colored as shown on the right side of the plots. RMSD and *R*_g_ are selected as the reaction coordinates. The structures in blue are taken from the vicinity of the global minimum.

**Fig 4:**
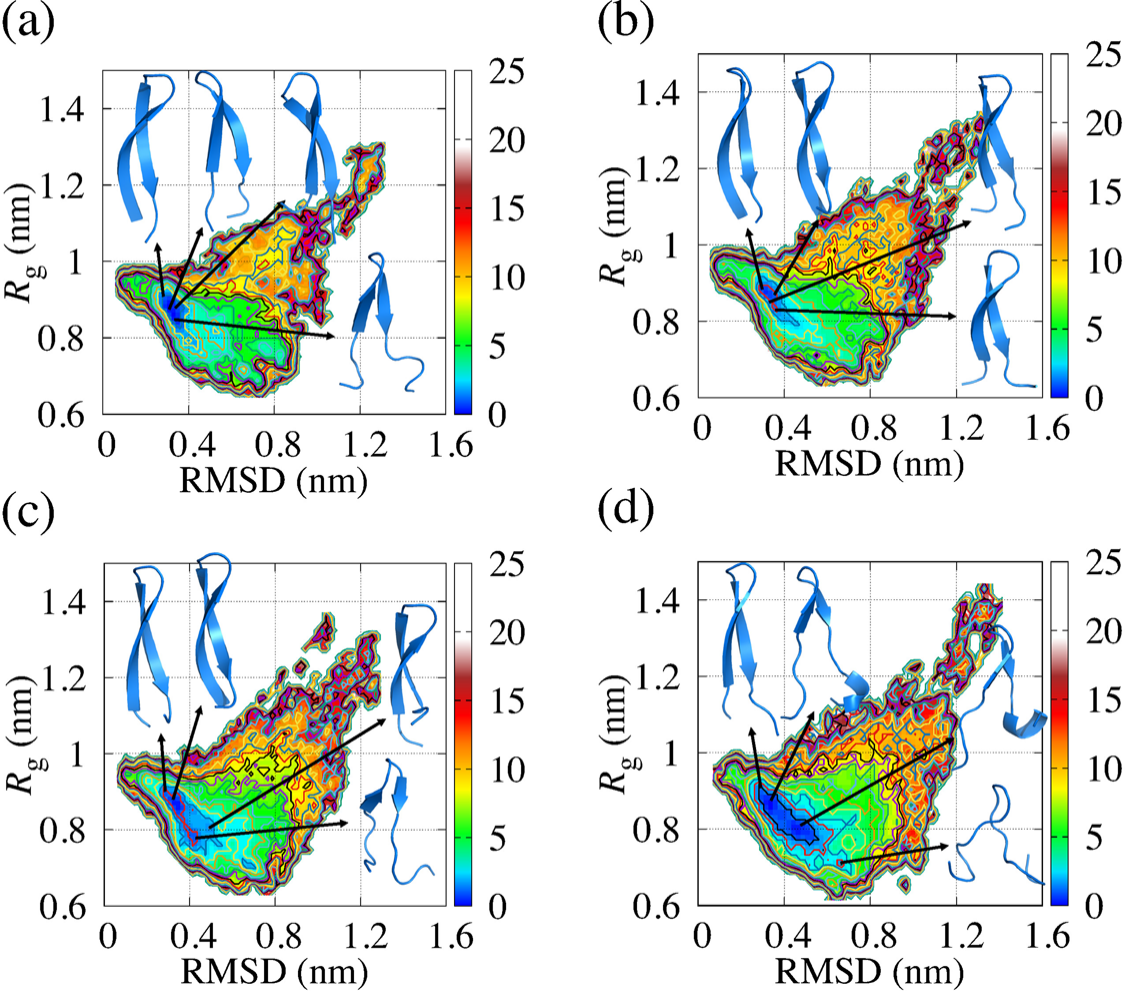
Free energy landscape of EF2 obtained from REMD. These maps indicate the existence probabilities of the EF2 peptide obtained from simulations at 300 K (a), 310 K (b), 322.5 K (c) and 343 K (d). Relative free energy change (kJ/mol) is colored as shown on the right side of the plots. Reaction coordinates are RMSD and *R*_g_. The structures in blue are selected from typical structures among conformations at the vicinity of the global minimum.

We mapped their free energy landscapes from the simulations. The root mean square deviation (RMSD) and radius of gyration (*R*_g_) were adopted as the reaction coordinates of the landscapes, which denote the variability of structures. We used the crystal structure as the reference structure for calculating the RMSD. We defined “the vicinity of the global minimum” as the region where Δ*G* is equal to or less than approximately 1.5 kJ/mol. In Fig 3A–3D, we showed the free energy landscape of EF1 at 300 K, 310 K, 322.5 K and 343 K. The structures taken from the vicinity of the global minimum showed β-sheet as a secondary structure. The free energy landscapes of EF2 and structures selected from the vicinity of the global minimum are shown in Fig 4A–4D. EF2 also had a hairpin-like structure around the global minimum, and the structures have β-strands as a secondary structure. The structural variation around the global minimum was observed in both EF1 and EF2. However, the structural variation is greater for EF2 than for EF1. We found that the structures have α-helix as a secondary structure in C-terminal. We also define “around the bottom of the global minimum” as the region where Δ*G* is equal to or less than approximately 2.5 kJ/mol. We showed the range of RMSD and *R*_g_ for the region around the global minimum of EF1 and EF2 in Table 5. The region around the bottom of the global minimum was broader for EF2 than for EF1 in most cases. We found that the structure of EF2 unfolded more easily than the structure of EF1 in the global minimum, suggesting that EF2 had some structural variability in the global minimum and easily escaped from the bottom of the free energy landscape compared with EF1.

**Table 5:** The range of RMSD and *R*_g_ for the region around the global minimum.

	Temperature (K)	RMSD (nm)	*R*_g_ (nm)
EF1	300	0.404–0.539	0.702–0.840
	310	0.368–0.602	0.690–0.850
	322.5	0.331–0.613	0.696–0.863
	343	0.287–0.607	0.688–0.880
EF2	300	0.300–0.659	0.719–0.931
	310	0.283–0.468	0.791–0.916
	322.5	0.277–0.707	0.700–0.911
	343	0.261–0.667	0.706–0.917

We focused on the points where the free energy of EF1 and EF2 had high values on the free energy landscape. The unfolding of the peptide main chain occurred at the positions where both the RMSD and *R*_g_ had large values, where the free energies of EF1 and EF2 had high values on the free energy landscape. Unfolding was observed not only on the free energy landscape of EF2, but also on that of EF1. The structures at the region where the value of *R*_g_ is 1.0 nm or grater are more frequently found for EF2 than for EF1. We found that, with a rise in temperature, the probability of finding the trajectory in the region increased for EF1 and EF2. It was indicated that EF2 had the structural variations in room temperature.

### Structural analysis of EF1 and EF2 by conventional MD simulation

**Fig 5:**
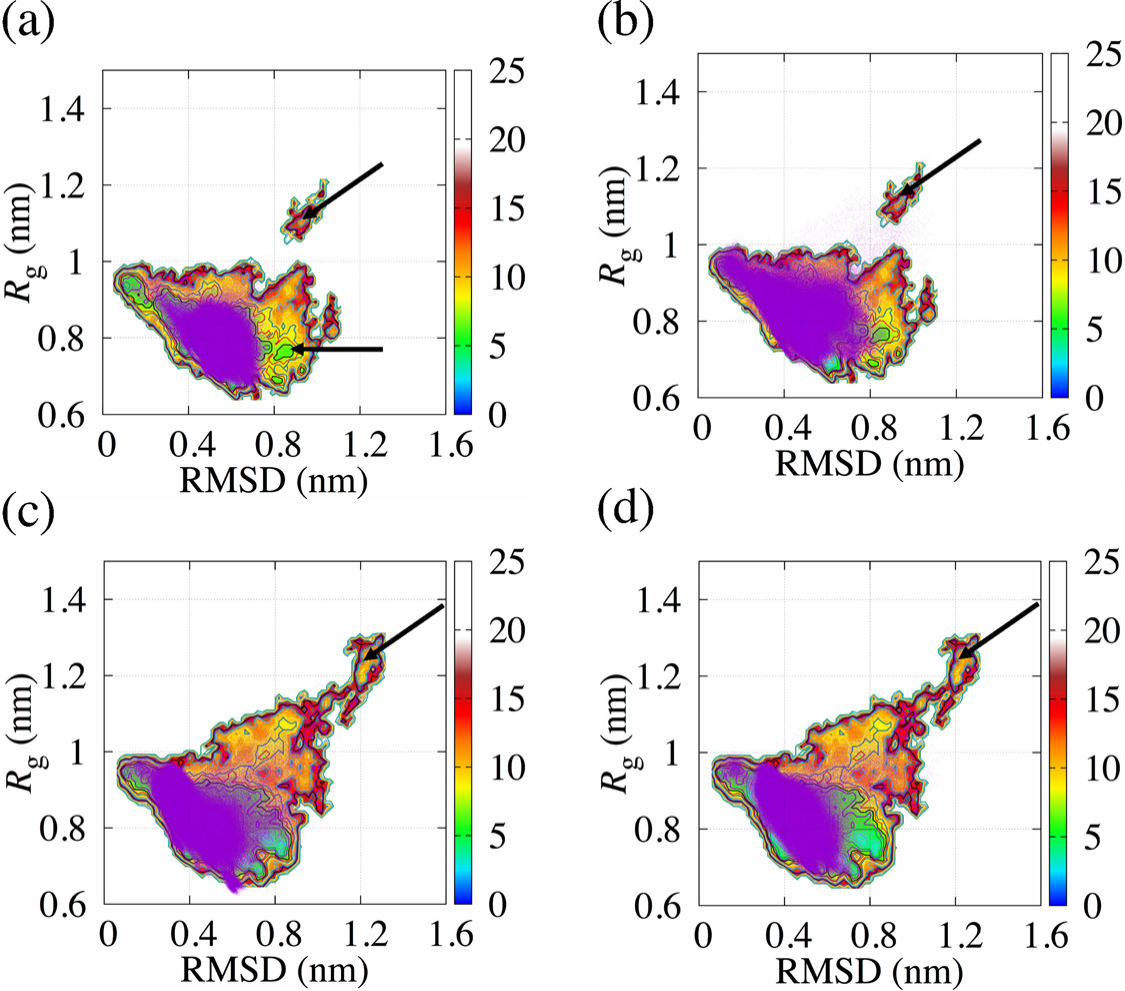
Trajectories of conventional MD simulations drawn on free energy landscape at 300 K obtained from REMD. Trajectories are plotted in purple on the free energy landscape. (a) and (b) correspond to two simulations of EF1, and (c) and (d) correspond to two simulations of EF2. Arrows indicate the areas where states were seldom observed in the time scale of the conventional MD.

Besides the REMD simulation described in the previous subsection, we performed conventional MD simulations for 2 μs at constant temperature and at constant pressure to investigate the dynamical behavior of EF1 and EF2. These conventional MD simulations were performed twice using different values of the initial position and the initial velocity of water molecules. To examine conformation changes in EF1 and EF2 from the crystal structure, we first calculated the RMSD of the main-chain (chiral carbon, nitrogen, and oxygen atoms) of EF1 and EF2 obtained from the simulations. The average values of RMSD and *R*_g_ are comparable for EF1 and EF2 as shown in Table 6, and the time evolution of RMSD and *R*_g_ are shown in S1 Fig and S2 Fig, respectively. To investigate how the free energy landscape obtained from REMD covered the trajectories of conventional MD simulation, we plotted the trajectories mapped on the coordinates of the RMSD and *R*_g_ with the landscapes as shown in Fig 5. The reaction coordinates of the trajectories of simulations of EF1 and EF2 change around the bottom of the free energy landscape. For EF1, in one of the two simulations, the trajectory was trapped at the region (local minimum A) including the global minimum as shown in Fig 5A. For EF1 and EF2, major structural changes did not occur in the time scale of the conventional MD simulation. The structures in the areas shown with arrows in Fig 5A–5D were seldom observed in the time scale of the conventional MD.

**Table 6:** Average values of RMSD and *R*_g_.

	RMSD	*R*_g_
EF1	0.531 (0.085) 0.455 (0.133)	0.793 (0.049) 0.826 (0.058)
EF2	0.454 (0.129) 0.445 (0.109)	0.829 (0.069) 0.845 (0.062)

Values in parentheses indicate the standard deviation.

**Fig 6:**
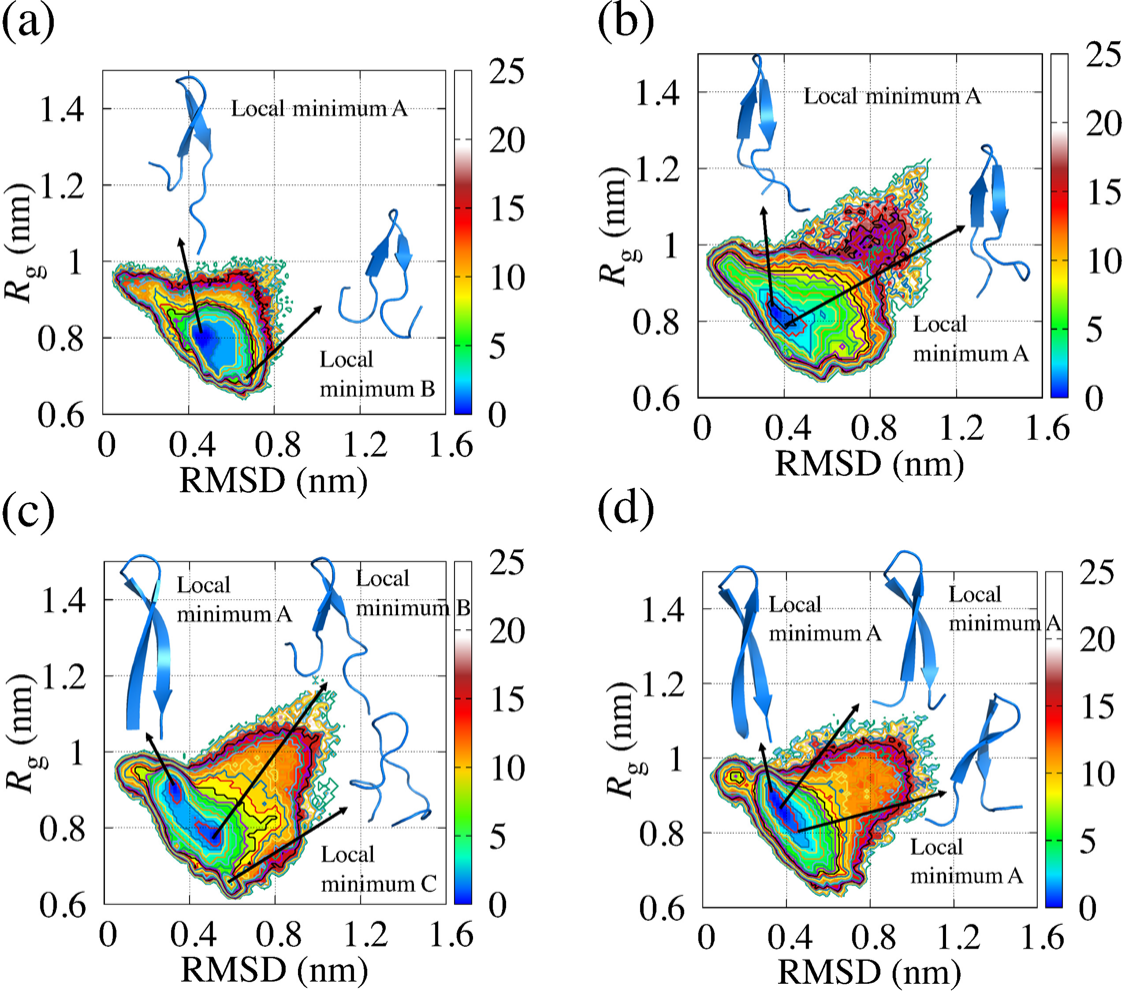
Free energy landscape of EF1 and EF2 obtained from conventional MD simulations. (a) and (b) are free energy landscapes of two simulations of EF1, and (c) and (d) are free energy landscapes of two simulations of EF2. Reaction coordinates are RMSD and *R*_g_. The displayed structures are selected from typical structures at local minima.

We have also drawn the free energy landscapes in order to obtain the local minimum structure of EF1 and EF2 during conventional MD simulations using the reaction coordinates RMSD and *R*_g_ as shown in Fig 6. For these maps, we found that EF1 had two local minima in Fig 6A and had a local minimum in Fig 6B. Δ*G* for the local minima is equal to or less than approximately 1.5 kJ/mol. EF2 had three local minima in Fig 6C and had a local minimum in Fig 6D. Among three local minima in Fig 6C, Δ*G* for the local minimum A and B is less than approximately 1.5 kJ/mol, and Δ*G* for the local minimum C is less than approximately 2.0 kJ/mol. The structural variation around the global minimum was observed in both EF1 and EF2 as with the free energy landscape obtained from REMD. Fig 6 displays the structures of EF1 and EF2 for the vicinity of the local minimum obtained from the free energy landscapes. The structure of EF1 for the local minimum had a hairpin-like structure for each run, and these structures included β-sheet as a secondary structure. In Fig 6B, the structure close to the crystal structure was often found. The differences among the RMSD of those structures were small, and the structures were similar. Thus, it was indicated that the β-sheet structure of EF1 would be maintained during the simulation. For the local minimum C in Fig 6C, we found that the values of *R*_g_ of the structures in the other local minima were different, and the difference between the RMSD of those structures was large. The structure of local minimum C is out of shape compared with the structures in the other local minima. These structures shown in Fig 6D had β-strand as a secondary structure. From these results, having defined “around the bottom of the global minimum” as the region where Δ*G* is equal to or less than 2.5 kJ/mol, it is suggested that the EF1 and EF2 fluctuated around the bottom of the global minimum obtained by REMD in the NPT ensemble for 2 μs.

**Fig 7:**
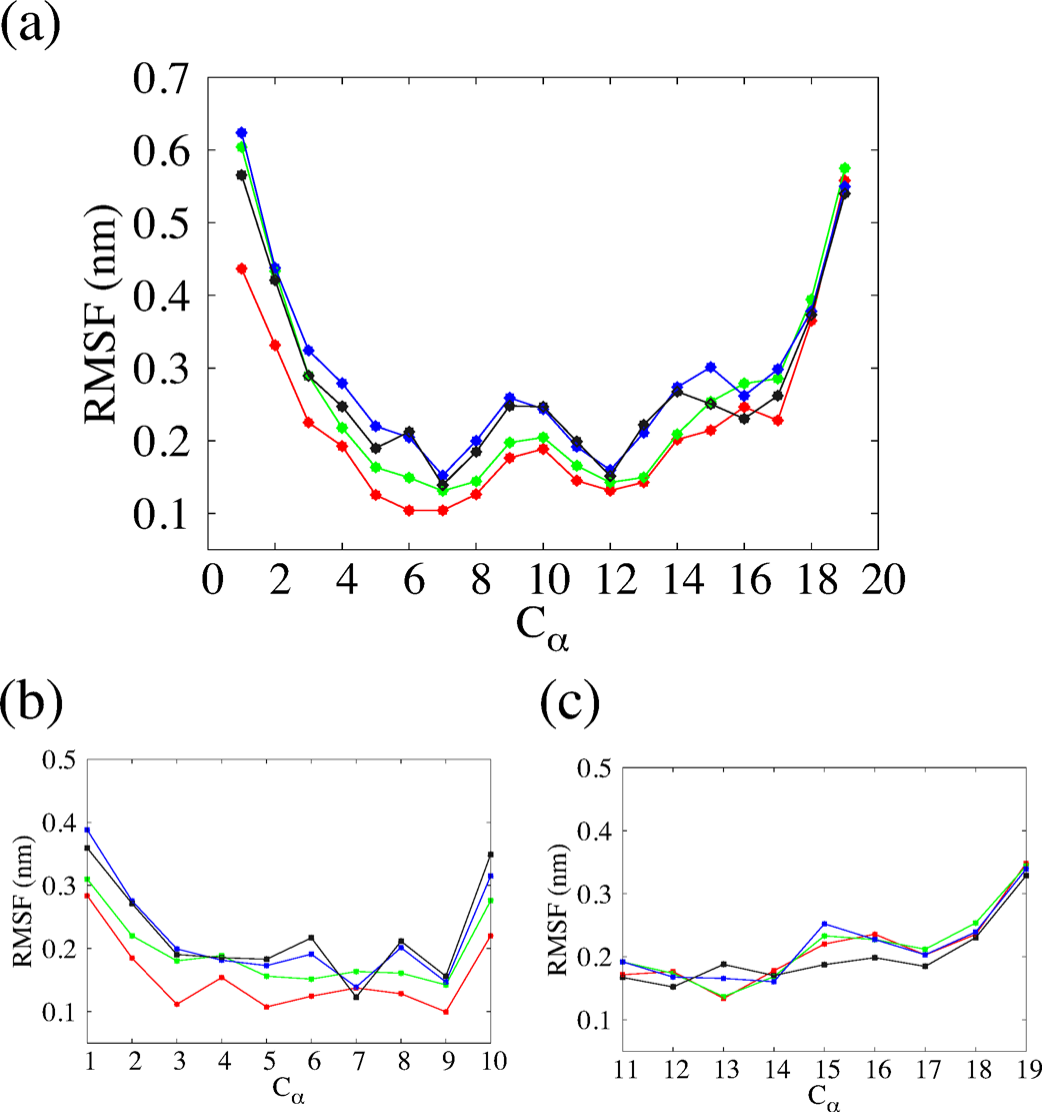
**Overall RMSF (C**_**α**_**) of EF1 and EF2 (a), RMSF (C**_**α**_**) for 1–10 residues (b), and RMSF (C**_**α**_**) for 11–19 residues (c).** Red and green lines correspond to two simulations of EF1, and black and blue lines correspond to two simulations of EF2.

It is necessary to understand the structural variation in the time evolution of EF1 and EF2 to further investigate the difference between the structures of EF1 and EF2. In order to investigate whether the fluctuation of each residue of EF1 and EF2 affects the structure of EF1 and EF2 during conventional MD simulations, we next calculated the root mean square fluctuation (RMSF) of EF1 and EF2. In Fig 7A, the overall RMSFs (C_α_, which is the chiral carbon in main chain) of EF1 and EF2 obtained by conventional MD simulations are shown. The fluctuations of the 4^th^ to 14^th^ residues of EF1 were smaller than those of EF2 for two simulations. We focused on residues whose fluctuations were large except for the terminals, and we observed that the 13^th^ to 16^th^ residues of EF1 fluctuate widely compared with the 4^th^ to 7^th^ residues. This means that the C-terminal of EF1 had larger fluctuation than the N-terminal. We then calculated the RMSF (C_α_) for the 1^st^ to 10^th^ residues (N side strand) and the 11^th^ to 19^th^ residues (C side strand) of EF1 and EF2. The plot in Fig 7B is different from the left half of Fig 7A because the reference system was obtained by fitting only the N side strand for Fig 7B. For one of the two simulations, the RMSF of the C side strand in EF1 was larger than that of the N side strand, and the result is consistent with the overall RMSF. We also found that the fluctuation of the N side strand of EF2 was larger than that of EF1. For C side strand, the fluctuations of EF1 and EF2 were similar to each other.

We also calculated the solvent accessible surface area (SASA) [48] as an index of structural change in EF1 and EF2 in conventional MD simulations. In general, the SASA for the structure of denatured states of peptide and protein is larger than that of the natural state. In Table 7, we show the SASA of EF1 and EF2 after energy minimization and the average values of the SASA of EF1 and EF2 obtained from conventional MD simulations. For EF1, the differences between the average value of SASA and the value of SASA after energy minimization are small, and the value of the difference was approximately 0.1 nm^2^. For EF2, the differences were larger than those of EF1, and the value of the difference is approximately 0.6–0.7 nm^2^. These results indicate that the structure of EF2 changed easily compared with the structure of EF1.

**Table 7:** Solvent accessible surface area (SASA) of EF1 and EF2.

SASA (nm^2^)	After energy minimization	Average values
EF1	7.992	7.896 (0.517) 7.913 (0.586)
EF2	7.695	8.307 (0.606) 8.418 (0.554)

Values in parentheses indicate the standard deviation.

### Non-covalent interactions

**Fig 8:**
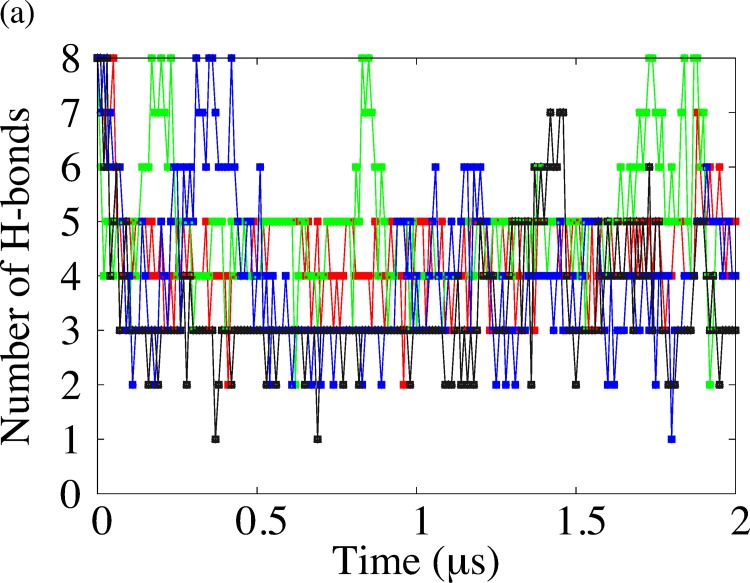
Number of H-bonds of EF1 and EF2 characterizing β-sheet structure. Red and green lines correspond to two simulations of EF1, and black and blue lines correspond to two simulations of EF2. The number of H-bonds is plotted every 10 ns.

**Fig 9:**
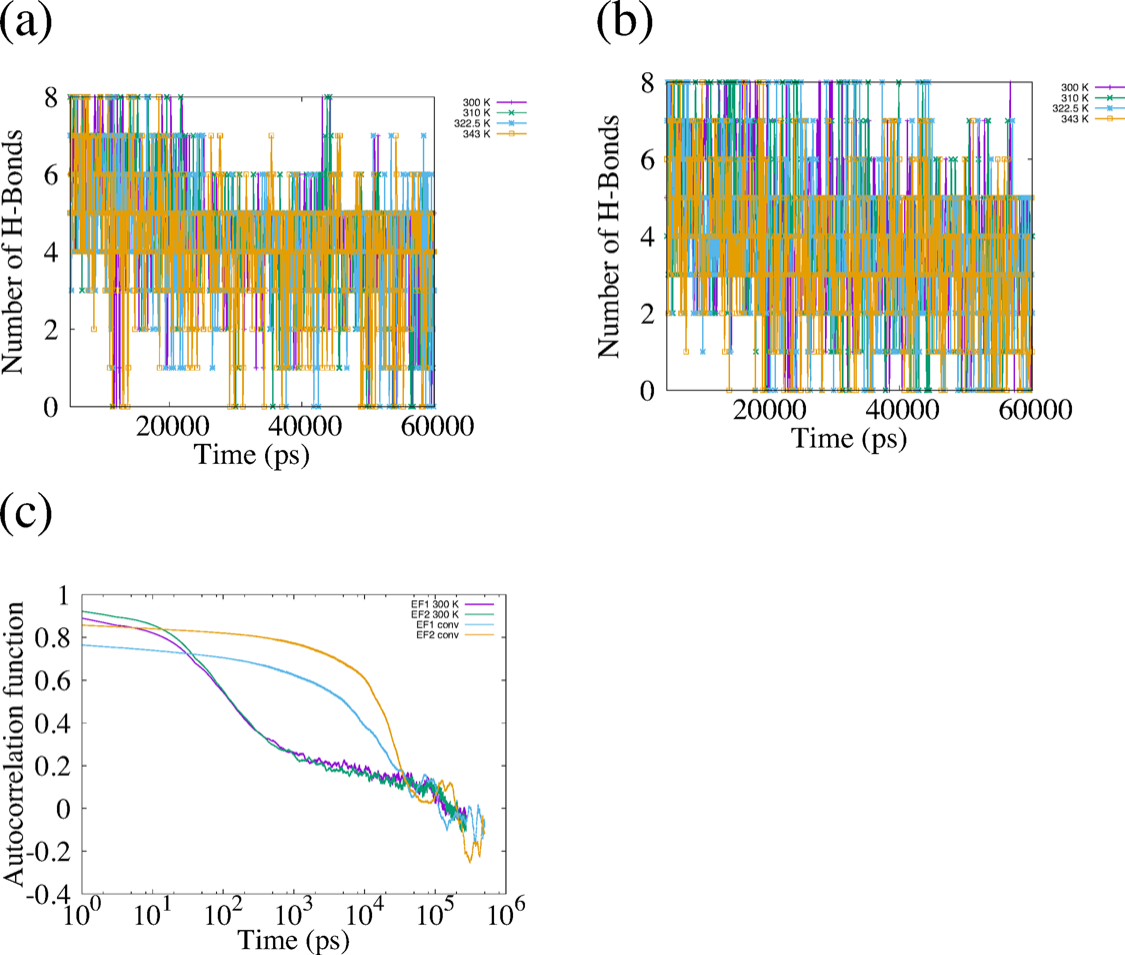
Number of H-bonds and autocorrelation function of the H-bonds. (a) and (b) indicate number of H-bonds of EF1 and EF2 for each replica (300 K, 310 K, 322.5 K and 343 K) used for analysis. The number of the H-bonds is plotted at every 1 ns. (c) is autocorrelation function of the number of the H-bonds obtained from REMD and the conventional MD. Purple and green colors are obtained from REMD at 300 K for REMD. Blue and yellow colors are obtained from the conventional MD.

**Fig 10:**
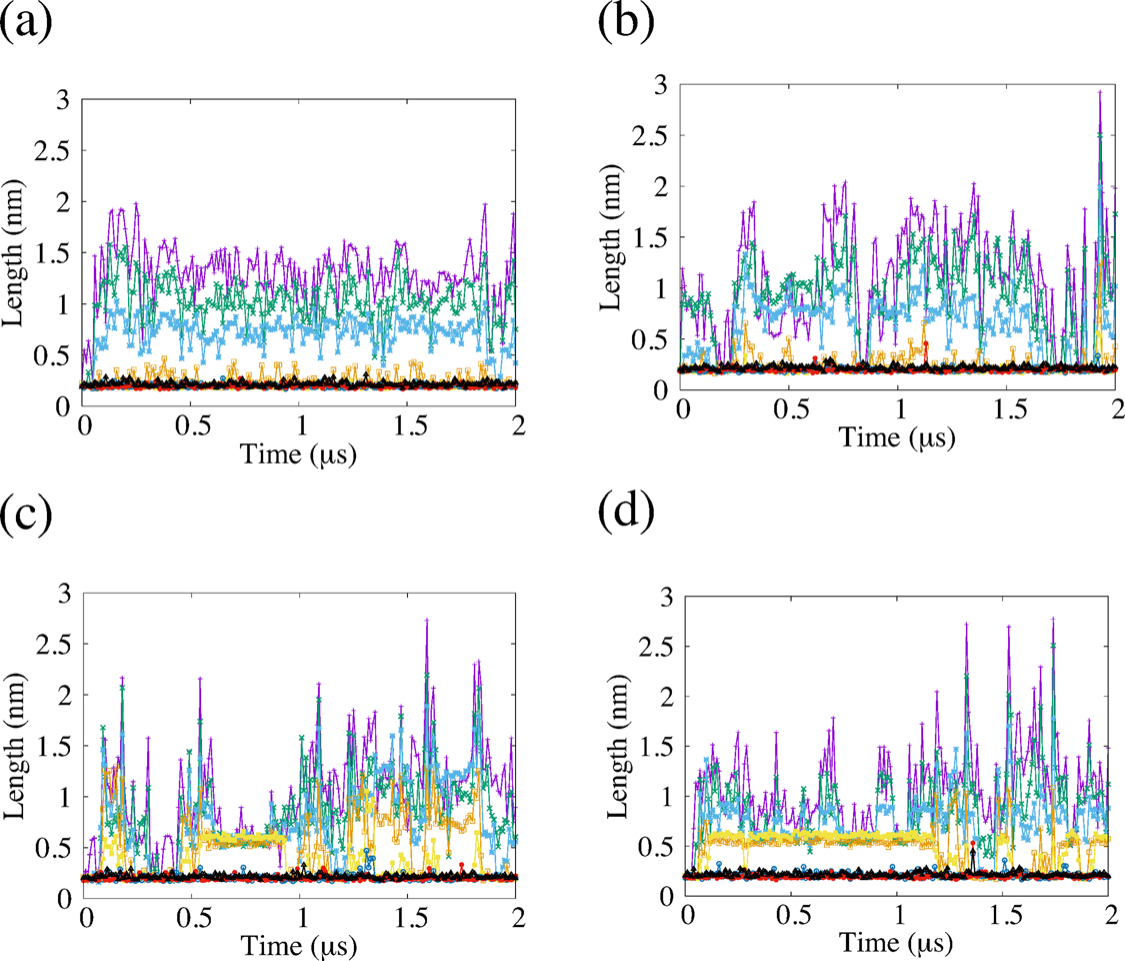
Length of hydrogen bonds (H-bonds), characterizing β-sheet structure of EF1 and EF2. The lines indicate the lengths of H-bonds for each pair of EF1 and EF2 shown in Table 3. (a) and (b) correspond to two simulations of EF1, and (c) and (d) correspond to two simulations of EF2. The length of H-bonds is plotted at every 10 ns.

In general, several types of non-covalent interactions, such as hydrophobic interactions, ionic interactions and hydrogen bonding, play important roles in forming and maintaining the folded structure of peptides and proteins. We calculate the lengths and the number of hydrogen bonds (H-bonds) in order to characterize the β-sheet structure. We display the time series of the number of H-bonds for each simulation in Fig 8 and the number of H-bonds and the standard deviation in Table 8. We observed that the number of H-bonds in EF1 was larger than that of EF2. We calculated the number and autocorrelation function of H-bonds for conformations obtained by REMD simulation for comparison and showed these quantities in Fig 9. The autocorrelation function of the number of H-bonds for REMD quickly decreased compared with the autocorrelation function for the conventional MD. We show the time series of each length (Fig 10) and the time–averaged length (Table 9) of H-bonds in EF1 and EF2, as shown in Table 3, for two simulations. Among eight H-bonds for EF1, five were maintained at a length of about 0.2–0.3 nm for two simulations. For the H-bonds in both runs of EF2, each length of the three H-bonds was maintained at about 0.2–0.3 nm. These maintained H-bonds were formed from β-turn side. These results show that the conformation of EF1 was more stable in the β-sheet structure than that of EF2.

**Table 8:** Number of H-bonds.

Values and standard deviation		
EF1	4.61 (0.80)	5.04 (1.15)
EF2	4.00 (1.45)	3.39 (1.18)

Values in parentheses indicate the standard deviation.

**Table 9:** Average distance of hydrogen bonds (H-bonds), characterizing β-sheet structure for EF1 and EF2.

	EF1_R1	EF1_R2	EF2_R1	EF2_R2
HB1	1.299 (0.302)	1.072 (0.505)	1.038 (0.459)	1.074 (0.397)
HB2	1.022 (0.252)	0.908 (0.399)	0.852 (0.403)	0.857 (0.337)
HB3	0.715 (0.171)	0.623 (0.298)	0.749 (0.396)	0.727 (0.249)
HB4	0.250 (0.070)	0.250 (0.121)	0.533 (0.305)	0.490 (0.188)
HB5	0.193 (0.140)	0.200 (0.053)	0.338 (0.184)	0.462 (0.186)
HB6	0.198 (0.016)	0.202 (0.029)	0.208 (0.038)	0.206 (0.025)
HB7	0.200 (0.023)	0.199 (0.024)	0.200 (0.023)	0.201 (0.030)
HB8	0.216 (0.023)	0.219 (0.025)	0.200 (0.023)	0.215 (0.025)

Values in parentheses indicate the standard deviation.

**Fig 11:**
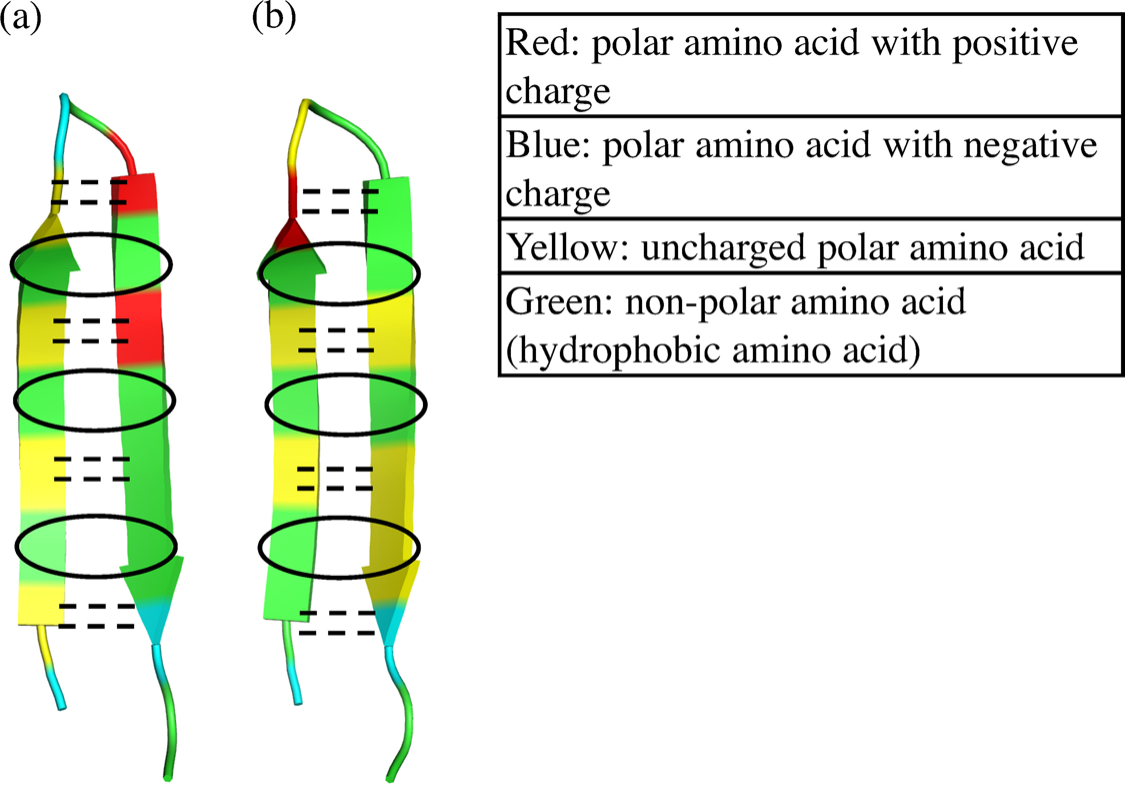
**Categorization of residues for (a) EF1 and (b) EF2 residues into four groups.** Dashed lines show positions of H-bonds. Open circles are the pairs of hydrophobic amino acids.

Next, we analyzed the results described above with polar and non-polar amino acid residues. In Fig 11, we categorized each residue in EF1 and EF2 into four types, which are (1) polar amino acid with positive charge, (2) polar amino acid with negative charge, (3) uncharged polar amino acid and (4) non-polar amino acid (hydrophobic amino acid). Hydrophobic interactions are attractive between hydrophobic amino acids in water solution. EF1 had three pairs of non-polar amino acids facing each other, and EF2 had two pairs. The pairs of H-bonds and the pairs of hydrophobic amino acids alternately appeared for EF1. In contrast, these pairs of amino acids in EF2 were not present at the terminals. In addition, EF1 had polar amino acids with both positive and negative charges in the center of the sequences, and there may be ionic interactions between these amino acids in EF1. The stability of HB4 and HB5 of EF1 is maintained by the hydrophobic interaction between ALA_3_ and PHE_14_ in sequences of EF1. On the other hand, the stability of HB4 and HB5 of EF2 is lost since EF2 does not have hydrophobic pair for the same positions in sequences of EF2. H-bonds, hydrophobic interactions, and ionic interactions may be related to the structural stability of EF1 and the fluctuation of EF2 in the terminals.

## Discussion

In our previous studies [35, 36], we performed simulated annealing [49] to investigate the structure for the global minimum of potential energy state of EF1 and EF2 peptides derived from the LG4 modules of laminin α1 and 2 chains. The results of simulated annealing show that hairpin-like structures were found for EF1, but not for EF2. Suzuki et al. suggested that the biological activity of EF1 depends on the hairpin-like structure [31], and our previous results were consistent with their experimental results. In this study, we performed structure sampling using the expanded ensemble to describe the free energy landscape which extensively covered the structures of EF1 and EF2. We also investigated the dynamical behavior of EF1 and EF2 peptides by performing conventional MD simulation.

We applied REMD simulations to EF1 and EF2 and efficiently sampled them, as shown in the free energy landscapes, compared with conventional MD simulations. For the free energy landscape of each replica used for analysis, the valley for EF2 was widespread on the free energy landscape compared with the valley for EF1. EF1 had β-sheet as a secondary structure in the global minimum, which is consistent with the results obtained from simulated annealing. EF2 had a variety of hairpin-like structures with β-sheet structure in the global minimum, as well as α-helix as a secondary structure, and it was found that the terminal of EF2 unfolded easily even in the global minimum because the region around the bottom of the global minimum of EF2 was wider than the region around the bottom of the global minimum of EF1 in most cases. It was indicated that the structure of EF1 with β-sheet is more stable than that of EF2 with β-sheet against small change of temperature. To analyze the dynamical behaviors of EF1 and EF2, conventional molecular dynamics simulations were performed twice in the NPT ensemble for 2 μs. To investigate the trajectories of conventional MD simulation on the free energy landscape obtained from REMD, we plotted the trajectories on the coordinates of the RMSD and *R*_g_ with the landscapes. We found that EF1 and EF2 fluctuate around the bottom of the free energy landscape. For EF1, the local minima of the free energy landscape shown in Fig 5A is trapped in the region, where the free energy is equal to or less than approximately 7.5 kJ/mol, including the global minimum. This result is consistent with the results obtained from REMD as the trajectories of EF1 were included around the bottom of the global minimum of the landscapes obtained from REMD. For the free energy landscape of EF2, the structure at the positions with a large RMSD and *R*_g_ (unfolding state) was observed. However, the range of value of *R*_g_ for the free energy landscapes for EF2 was wider than the range for EF1. The probability distributions were different between EF1 and EF2. The probability distributions of the region where value of *R*_g_ is 1.0 nm or greater on the free energy landscape for EF2 was larger than that for EF1. The structures in the regions shown with arrows in Fig 5A–5D were seldom observed by conventional MD simulation.

From REMD analysis, our data showed that the structural variability of EF1 was smaller than that of EF2, while EF1 and EF2 peptides fluctuated around the bottom of the global minimum of the free energy landscape in conventional MD simulations. We have drawn the free energy landscapes of EF1 and EF2 for each of two conventional MD simulations. For EF1, the local minima structures for each map were hairpin-like structures, with β-sheet as a secondary structure. For EF2, one of the local minima was a β-sheet structure. Thus, it is suggested that both EF1 and EF2 can have β-sheet structures in the time scale of the conventional MD simulations. For EF2, there was a difference between the RMSD values of the three local minima shown in Fig 6C, and the *R*_g_ of the structures were also different at the three local minima. On the free energy landscape, the distribution of EF1 was narrow, while that of EF2 was wide. Although the difference between the trajectory distributions of EF1 and EF2 shown in Fig 5 seemed to be small, the probability distributions were different between EF1 and EF2, as shown in Fig 6. To show the differences in trajectory distributions, it is important to calculate the probability distributions.

The conformational difference in EF2 is related to the fluctuation of each residue, and we calculated the RMSF for C_α_ in each residue of EF1 and EF2. We found that the fluctuation of EF2 was larger than that of EF1, and that the N-terminal (1^st^ to 10^th^ residues) of EF2 fluctuated markedly. From the results of SASA, the average value for EF1, which was about 0.1 nm^2^, was close to the value after energy minimization, and the average value for EF2 was about 0.6–0.7 nm^2^, which was much larger than the value after energy minimization. These differences between EF1 and EF2 affected the variations in their conformation, and the fluctuation in the N-terminal of EF2 may have affected the conformational variability. There was only a slight difference between the structures of EF1 obtained from the local minimum because the fluctuation of EF1 was small.

Since, from REMD simulation, the region around the bottom of the free energy landscapes was narrower for EF1 than for EF2, we can consider that EF2 unfolded easily compared with EF1, and that EF1 had stable β-sheet structure in water solution while the β-sheet structure of EF2 was unstable. In our preliminary study [50], we indicated that H-bonds were important for the structural maintenance of β-strands. The importance of H-bonds for β-strand peptides was also pointed out in Hatfield’s paper [51], involving a molecular dynamics analysis of CLN025 (a stabilized Chignolin miniprotein). Csontos et al. [52] also reported that the structure of tripeptides with aromatic backbone is stabilized by H-bonds, indicating that H-bonds are important for structural stabilization. Several groups [53–56] have studied the free energy landscape with the number of H-bonds and radius of gyration for other peptides and proteins. To investigate whether β-sheet is maintained during the conventional MD simulations, we measured the average length and the number of H-bonds characterizing β-sheet structure. In this study, we focused on eight H-bonds between N-H and C = O in peptide bonds of adjacent main chains, and we confirmed that the number of H-bonds in EF1 was larger than that of EF2. Among eight H-bonds shown in Table 3, the maintenance of the H-bonds at HB4 and HB5 is especially important. For EF2, it is considered that the average value of SASA was larger than that after the energy minimization when the number of H-bonds was small. This relationship between H-bonds and the value of SASA for amyloidogenic fragments using compartments (15–19 residues) of Islet Amyloid Polypeptide (IAPP) was reported by Singh’s paper [57]. We also observed that the magnitude of the fluctuation of EF1 in the terminals was smaller than that of EF2. These results show that H-bonds were maintained for EF1, while not maintained for EF2.

In addition to H-bonds, we considered other non-covalent interactions from amino acid sequences of EF1 and EF2. EF1 has three pairs of residues with hydrophobic interaction, and EF2 has two pairs. Furthermore, EF1 may have ionic interaction between these amino acids because it has polar amino acids with positive and negative charges in the center of the sequences. EF1 has eight pairs of non-covalent interactions, while EF2 has six pairs of non-covalent interactions. EF2 is considered to fluctuate strongly because of the absence of the pair of hydrophobic amino acids between both terminals. These factors may be related to the difference in fluctuation and structural variations between EF1 and EF2. The stability of H-bonds at HB4 and HB5 are especially enhanced by the hydrophobic interaction between ALA_3_ and PHE_14_.

From Katagiri’s experiments [33], EF1 showed cell attachment activity, whereas EF2 did not. Suzuki et al. also showed that EF1 has cell attachment activity [31], and they reported the importance of the hairpin-like structure. In conclusion, it is suggested that the suppression of structural fluctuation around N-terminal and C-terminal is important to maintain β-sheet structure (the hairpin-like structure). Since it is important that several non-covalent interactions are formed for structural stabilization, we propose that both of H-bonds in the main chain and the pair of hydrophobic amino acids in the terminals are important in stabilizing the structure. We hope that our study, which is the conformation analysis of cell attachment peptides derived from laminin, will be useful for future studies in the application of laminin in such areas as medicine and biomaterials.

## Supporting Information

S1 Fig RMSD values of EF1 and EF2 in conventional MD. (a) and (b) show the time evolution of RMSD for EF1 and for EF2. Red and blue lines correspond to two simulations of EF1, and green and yellow lines correspond to two simulations of EF2.
(PDF)

S2 Fig *R*_g_ values of EF1 and EF2 in conventional MD. (a) and (b) show the time evolution of *R*_g_ for EF1 and for EF2. Red and blue lines correspond to two simulations of EF1, and green and yellow lines correspond to two simulations of EF2.
(PDF)
