# Supplementary figures and images for: Structural Study of Cell Attachment Peptide Derived from Laminin by Molecular Dynamics Simulation
Source: PLoS One. 2016 Feb 18;11(2):e0149474. doi: 10.1371/journal.pone.0149474 (PMC4759364; doi:10.1371/journal.pone.0149474)

(a)

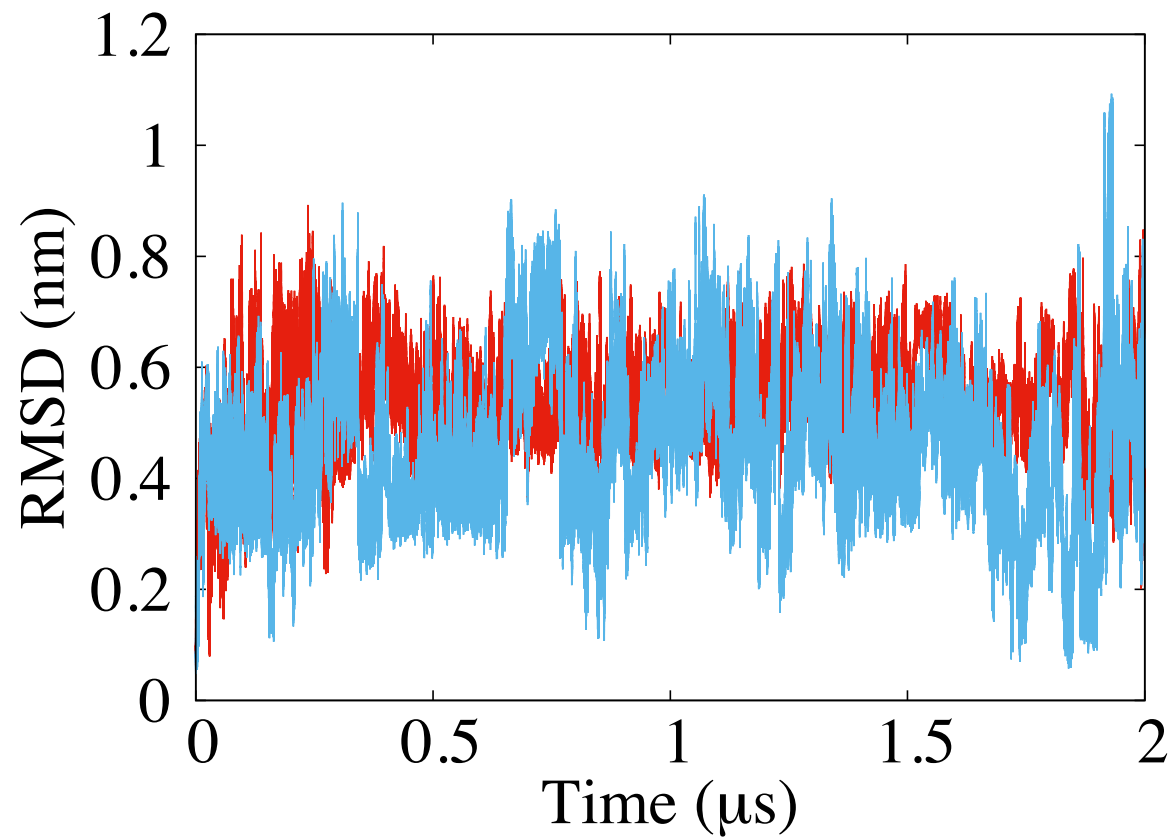

(b)

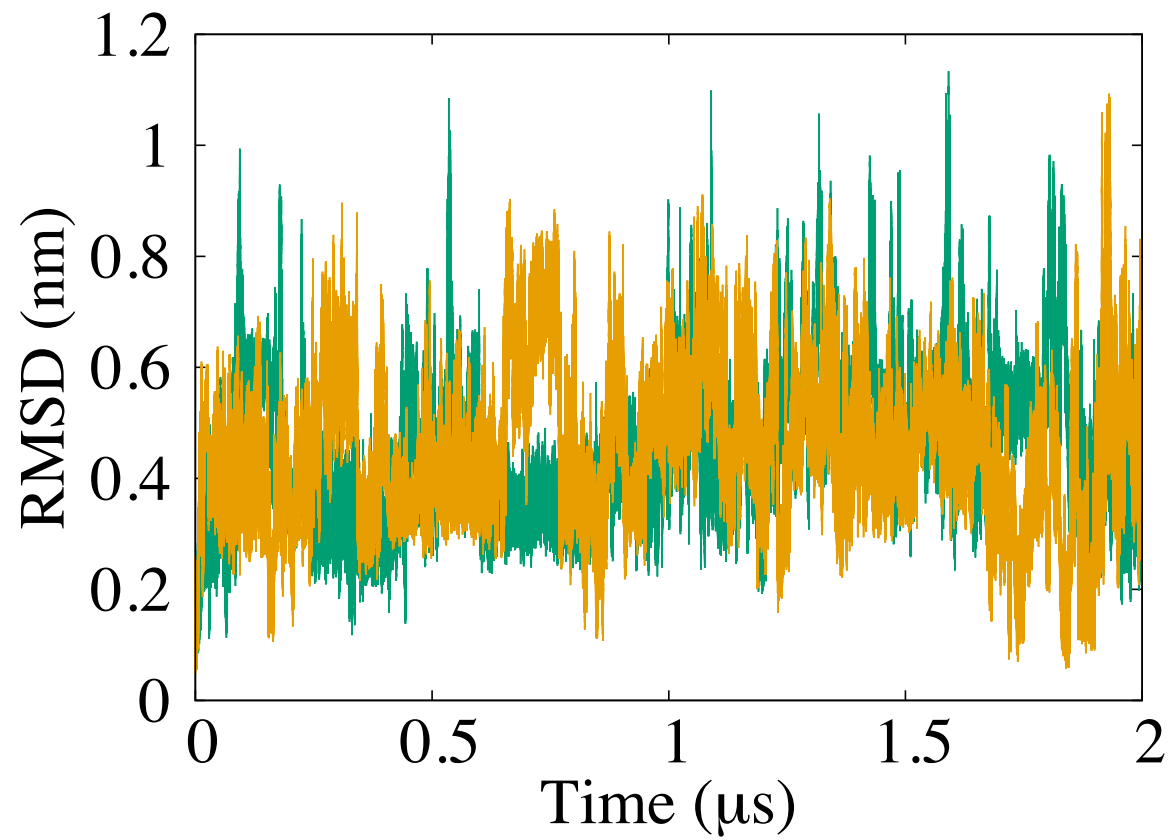

Supplement: S1 Fig — (a) and (b) show the time evolution of RMSD for EF1 and for EF2. Red and blue lines correspond to two simulations of EF1, and green and yellow lines correspond to two simulations of EF2. (PDF) [file pone.0149474.s001.pdf]

(a)

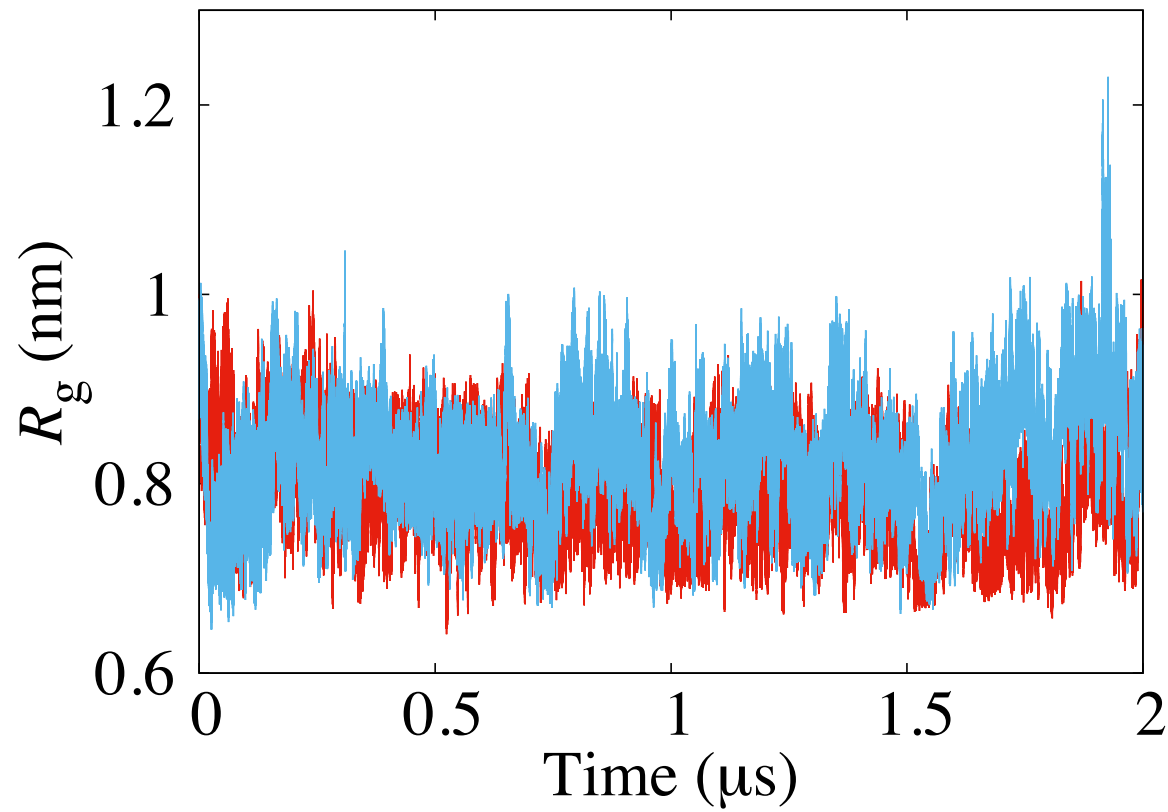

(b)

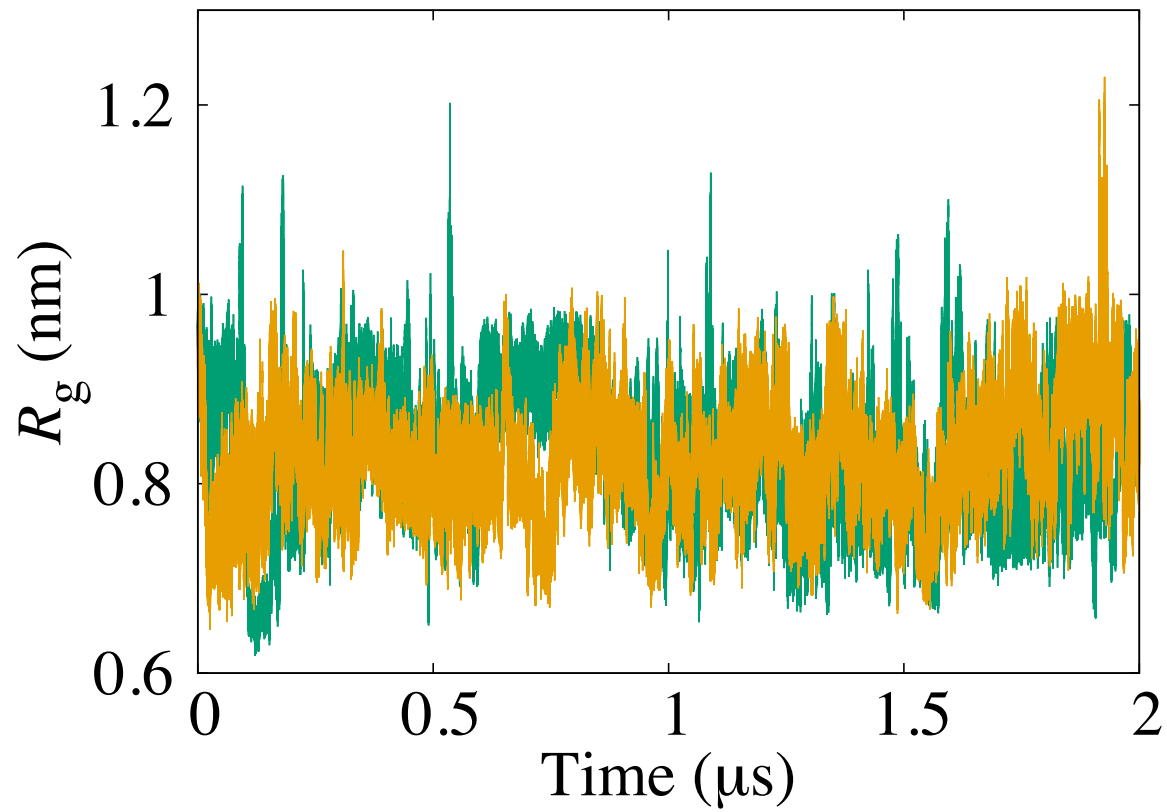

Supplement: S2 Fig — (a) and (b) show the time evolution of Rg for EF1 and for EF2. Red and blue lines correspond to two simulations of EF1, and green and yellow lines correspond to two simulations of EF2. (PDF) [file pone.0149474.s002.pdf]
